# Association of prostate‐specific antigen density with prostate cancer mortality after a benign systematic prostate biopsy result

**DOI:** 10.1111/bju.16641

**Published:** 2025-01-22

**Authors:** Juho Pylväläinen, Kirsi Talala, Jani Raitanen, Antti Rannikko, Anssi Auvinen

**Affiliations:** ^1^ Department of Radiology, HUS Diagnostic Centre Helsinki University Hospital Helsinki Finland; ^2^ Department of Urology Helsinki University Hospital Helsinki Finland; ^3^ Research Program in Systems Oncology, Faculty of Medicine University of Helsinki Helsinki Finland; ^4^ Cancer Society of Finland Helsinki Finland; ^5^ Faculty of Social Sciences (Health Sciences), Prostate Cancer Research Center Tampere University Tampere Finland; ^6^ UKK Institute for Health Promotion Research Tampere Finland

**Keywords:** comparative study, follow‐up, prostate biopsy, prostate cancer, prostate‐specific antigen density

## Abstract

**Objective:**

To assess the association between prostate‐specific antigen (PSA) density (PSAD) and prostate cancer mortality after a benign result on systematic transrectal ultrasonography (TRUS)‐guided prostate biopsy.

**Patients and Methods:**

This retrospective study used data from the Finnish Randomised Study of Screening for Prostate Cancer (FinRSPC) collected between 1996 and 2020. We identified men aged 55–71 years randomised to the screening arm with PSA ≥4.0 ng/mL and a benign systematic TRUS‐guided biopsy result. The cumulative prostate cancer mortality of men stratified by a PSAD cutoff of 0.15 ng/mL/cm^3^ was modelled with competing risk functions. The ability of PSAD, PSA, and base variables (age at biopsy, DRE result, socioeconomic status, 5α‐reductase inhibitor usage, family history, and Charlson Comorbidity Index (CCI)) to predict prostate cancer death was compared using *c*‐statistics and a likelihood ratio test.

**Results:**

After excluding 10 men without PSA data within 2 years of the biopsy and 65 without prostate volume data, 2276 men were eligible for inclusion in the study. A total of 50 men died from prostate cancer and 1028 from other causes during a median (interquartile range) follow‐up of 17.4 (13.2–20.9) years. The cumulative prostate cancer mortality of men with PSAD <0.15 ng/mL/cm^3^ was significantly lower than that of men with PSAD ≥0.15 ng/mL/cm^3^: 0.5% (95% confidence interval [CI] 0.2%–1.1%) vs 2.0% (95% CI 1.2%–3.1%) at 15 years (Grey's test, *P* = 0.001). The model consisting of PSAD, PSA and the base variables predicted prostate cancer mortality (*c*‐statistic 0.781) significantly better than either the base variables alone (*c*‐statistic 0.737; likelihood‐ratio test, *P* = 0.003) or the base variables and PSA (*c*‐statistic 0.765; likelihood‐ratio test, *P* = 0.039).

**Conclusion:**

Prostate cancer mortality after a benign systematic TRUS‐guided biopsy is low. In these patients, PSAD predicts prostate cancer mortality and provides additional value to other clinical variables. PSAD‐based stratification can be used to guide follow‐up strategy.

**Abbreviations**
5‐ARI: 5α reductase inhibitor
AIC: Akaike information criterion
CCI: Charlson Comorbidity Index
ERSPC: European Randomised Study of Screening for Prostate Cancer
FinRSPC: Finnish Randomised Study of Screening for Prostate Cancer
IQR: interquartile range
LOESS: locally estimated scatterplot smoothing
PSAD: PSA density
TRUS: transrectal ultrasonography

## Introduction

Early detection of prostate cancer using DRE and PSA testing with subsequent systematic transrectal ultrasonography (TRUS)‐guided biopsy or MRI is the current standard of care [1]. However, approximately 40% of men undergoing conventional TRUS‐guided biopsy and 30% of men who undergo prostate MRI have a benign result [2, 3]. The risk of harbouring Gleason score ≥7 prostate cancer and subsequent disease‐specific death after a benign result on systematic TRUS‐guided biopsy are low but not negligible [2, 3, 4, 5, 6]. While MRI is better than a systematic TRUS‐guided biopsy at excluding Gleason score ≥7 prostate cancer (91% vs 86%), its association with disease‐specific mortality remains unclear due to its relative novelty [3]. As neither conventional TRUS‐guided biopsy nor prostate MRI can exclude clinically significant prostate cancer, men with a benign result are generally followed with PSA testing, repeat MRI scans, and MRI‐targeted or systematic TRUS‐guided biopsies. The strategy aims to ensure diagnosis of an initially missed clinically significant prostate cancer but predisposes the men to unnecessary biopsies and overdiagnosis.

Patient stratification with reflex tests, such as PSA density (PSAD), can help mitigate monitoring‐related harms [7]. PSAD has been developed as an improvement on PSA, which can predict long‐term risk of Gleason ≥7 prostate cancer [8] and disease‐specific mortality [9, 10] but is also notorious for its low specificity. As PSAD is defined as the plasma PSA concentration divided by the prostate volume, it inherently accounts for hyperplasia, which is the most common benign cause of elevated PSA [7, 11]. While PSAD has been shown to outperform PSA in predicting subsequent prostate cancer, its association with prostate cancer mortality has not been demonstrated at population level [7, 12].

This study aims to assess prostate cancer‐specific mortality in relation to PSAD in men with a benign systematic TRUS‐guided prostate biopsy result and to determine if PSAD can improve prediction of prostate cancer death in addition to total PSA.

## Patients and Methods

### Study Design and Population

This was a retrospective cohort study that used data from the Finnish Randomised Study of Screening for Prostate Cancer (FinRSPC) [13], the largest component of the European Randomised Study of Screening for Prostate Cancer (ERSPC; https://www.erspc.org/prostate‐cancer/erspc‐background/), a multicentre screening trial which evaluated the effectiveness of PSA‐based prostate cancer screening. The study protocol has been described previously [13]. In brief, of 80 458 men born in 1929–1944 (aged 55, 59, 63 and 67 years at entry) identified from the Finnish Population Registry, 8000 were allocated to the annual screening arm of the study in 1996–1999. These men were invited to provide a blood sample for measuring serum PSA concentration. Men with PSA levels ≥4.0 ng/mL were referred to a local urological clinic for diagnostic assessment, including DRE and systematic TRUS‐guided biopsy. For men with a serum PSA level of 3.0–3.9 ng/mL, a prostate biopsy was recommended if there was a suspicious DRE finding (in 1996–1998) or if the proportion of free PSA was <0.16 (after 1999), but we excluded such men from the analysis. Sextant biopsies were used initially; however, 10–12 biopsy cores were obtained from 2002 onwards. The men in the screening arm were invited to new rounds of similar screening at 4 and 8 years after the initial screening, up to the age of 71 years.

For this study, we included all men in the screening arm who on any screening round had a PSA level ≥4.0 ng/mL and who subsequently underwent a systematic TRUS‐guided biopsy, with a benign result. We collected information from the medical records on age at biopsy, DRE result, total serum PSA concentration prior to the biopsy, 5α reductase inhibitor (5‐ARI) usage during the year of PSA measurement, the estimated prostate volume on TRUS, and Gleason score at biopsy (Table 1). We identified socioeconomic status, date and cause of death (prostate cancer vs other causes) through Statistics Finland. We also collected data on self‐reported family history of prostate cancer. Prostate volume was measured using ellipsoid estimation based on the width, height and length of the prostate. Information on prostate cancer diagnosis was obtained from the nationwide population‐based Finnish Cancer Registry, with 96% completeness for solid cancers (available only until 31 December 2016) [14]. We estimated comorbidity burden at biopsy using Deyo's adaption of the Charlson Comorbidity Index (CCI) [15], calculated from the Finnish National Care Register for Health Care [16] (data availability limited to 1996–2015) using a previously validated method [17]. Collection of comorbidity data was limited to up to 4 years prior to the biopsy.

**Table 1:** Clinical and background characteristics of participants stratified by PSA density.

Variables	PSAD <0.15 ng/mL/cm^3^ (*n* = 1367)	PSAD ≥0.15 ng/mL/cm^3^ (*n* = 909)	*P* value*
Age at initial biopsy, median (IQR), years	64.0 (62.7–67.5)	63.3 (59.4–67.1)	<0.001
Follow‐up, median (IQR), months	211 (164–250)	213 (156–261)	0.3
PSA, median (IQR), ng/mL	4.88 (4.38–5.74)	6.42 (5.16–8.65)	<0.001
PSAD, median (IQR), ng/mL/cm^3^	0.11 (0.09–0.13)	0.20 (0.17–0.26)	<0.001
Prostate volume, median (IQR), cm^3^	49 (40–60)	31 (26–39)	<0.001
PSA to biopsy difference, median (IQR), days	65 (53–80)	59 (49–73)	<0.001
CCI score, *n* (%)	*n* = 1367	*n* = 909	0.10
0	1261 (92)	840 (92)	
1	69 (5.0)	50 (5.5)	
2	33 (2.4)	12 (1.3)	
3+	4 (0.3)	7 (0.8)	
Family history of prostate cancer, *n* (%)	*n* = 1364	*n* = 905	0.7
No family history	1248 (91)	824 (91)	
First‐degree relative with prostate cancer	116 (8.5)	81 (9.0)	
Unknown	3	4	
Socioeconomic class, *n* (%)	*n* = 1340	*n* = 893	0.4^†^
Unemployed	137 (10)	101 (11)	
Manual worker	139 (10)	107 (12)	
Lower level employees	144 (11)	99 (11)	
Self‐employed person	70 (5.2)	58 (6.5)	
Upper level employees	220 (16)	138 (15)	
Pensioner	630 (47)	392 (44)	
Unknown	27	16	
DRE result, *n* (%)	*n* = 1330	*n* = 882	<0.001
Normal	1210 (91)	758 (86)	
Suspicious	119 (8.9)	122 (14)	
Malignant	1 (<0.1)	2 (0.2)	
Unknown	37	27	
Biopsy cores, *n* (%)	*n* = 1367	*n* = 909	<0.001
2–6	903 (66)	698 (77)	
7–10	94 (6.9)	70 (7.7)	
11–12	367 (27)	140 (15)	
>12	3 (0.2)	1 (0.1)	
5‐ARI usage, *n* (%)	107 (7.8)	33 (3.6)	0.004
Screening round at biopsy, *n* (%)	*n* = 1367	*n* = 909	<0.001
First	601 (44)	537 (59)	
Second	478 (35)	253 (28)	
Third	288 (21)	119 (13)	
Re‐biopsy, *n* (%)	689 (50)	571 (63)	<0.001
Prostate cancer status^‡^, *n* (%)	*n* = 1367	*n* = 909	<0.001
Free of disease	1046 (77)	524 (58)	
Gleason score ≥7	104 (7.6)	132 (15)	
Gleason score ≤6	166 (12)	221 (24)	
Unknown grade	51 (3.7)	32 (3.5)	
Primary outcome, *n* (%)	*n* = 1367	*n* = 909	0.001
Alive	732 (54)	458 (50)	
Emigrated	5 (0.4)	3 (0.3)	
Other‐cause death	612 (45)	416 (46)	
Prostate cancer death	18 (1.3)	32 (3.5)	
Age at prostate cancer‐specific death, median (IQR), years	79 (77–86)	79 (74–83)	0.11
Age at other‐cause death, median (IQR), years	79 (75–84)	77 (72–82)	<0.001
Latency of prostate cancer diagnosis to disease specific death, median (IQR), months	66 (34–126)	115 (69–164)	0.10
Latency of initial biopsy to disease specific death, median (IQR), months	189 (177–223)	170 (126–204)	0.11
Prostate cancer diagnosis prior to disease‐specific death^‡^, *n* (%)	*n* = 18	*n* = 32	0.3
Gleason score ≥7	14 (78)	20 (63)	
Gleason score ≤6	3 (17)	10 (31)	
No diagnosis	1 (5.6)	0 (0)	
Unknown grade	0 (0)	2 (6.3)	
Follow‐up ≥15 years, *n* (%)	1231 (90)	854 (94)	0.004

5‐ARI, 5α‐reductase inhibitor; CCI, Charlson Comorbidity Index; IQR, interquartile range; PSAD, PSA density.

* Mann–Whitney *U*‐test; Pearson's chi‐squared test; Fisher's exact test.

^†^ Calculated when ‘Student’ treated as missing data.

^‡^ Prostate cancer grade was determined at the time of diagnosis and may not represent the highest grade subsequently identified.

We excluded men with a previous prostate cancer diagnosis, missing prostate volume or prostate volume larger than 300 mL, or lacking PSA values within a period from 2 years prior to until 30 days after the initial biopsy.

The primary endpoint was prostate cancer mortality and the secondary endpoint was other‐cause mortality. Follow‐up started from the date of the initial biopsy and ended on the closing date of the study on 31 December 2020, death, or emigration. Participants who emigrated, died from other causes, or were alive at the end of our study were right censored (Table 1). Findata (THL/1182/14.06.00/2021) and Statistics Finland (TK/1316/07.03.00/2023, U0217_jl5) granted research permissions. The original study protocol was approved by the Ethics Committee of the Pirkanmaa Hospital District (tracking number R10167). The study was conducted in compliance with the STROBE guidelines for observational studies and in compliance with the good research practices of the World Medical Association Declaration of Helsinki. The data were handled in accordance with national laws and European Union regulations.

### Statistical Analysis

To assess the association of PSAD with prostate cancer mortality, we stratified the study population by PSAD using a commonly applied threshold of 0.15 ng/mL/cm^3^ [18]. The clinical characteristics of these two subgroups are shown in Table 1. Differences between the PSAD strata were assessed using the Mann–Whitney *U*‐test for continuous variables and Fisher's exact test and Pearson's chi‐squared test for categorical variables.

**Fig. 1:**
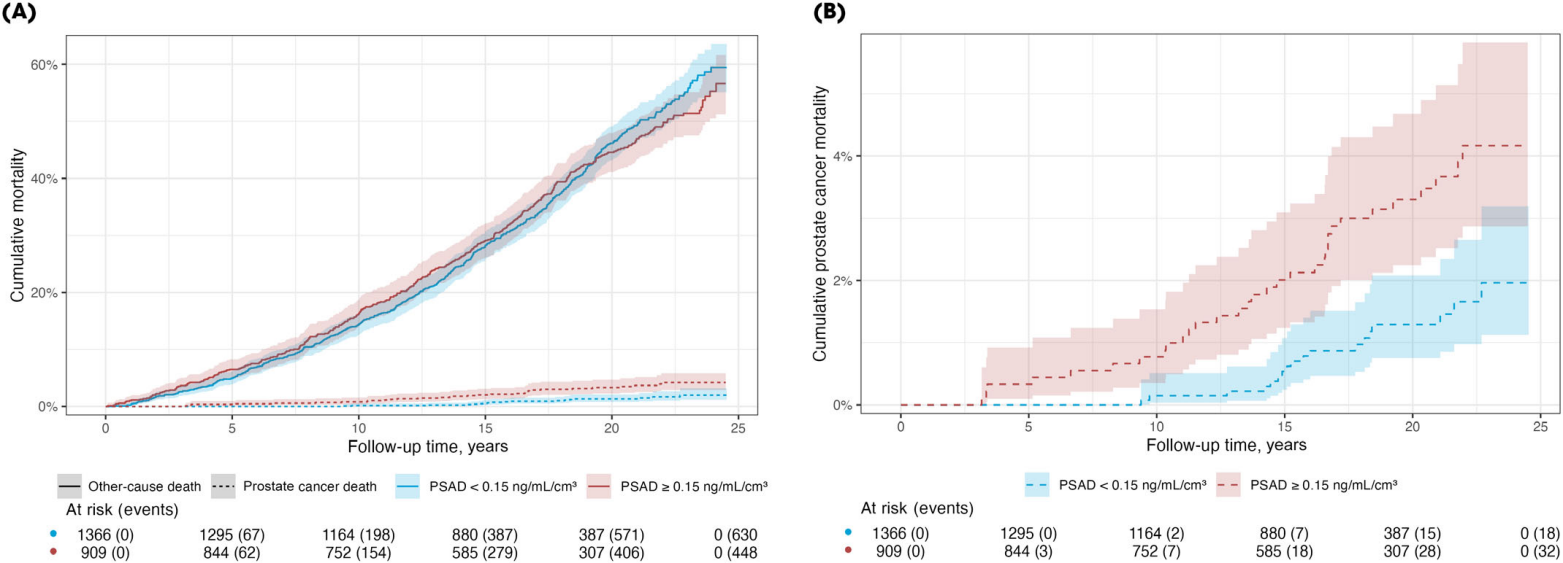
Plot illustrating the cumulative incidence of other‐cause death (solid line) and prostate cancer death (dashed line) following a benign systematic TRUS‐guided biopsy result in men with PSA density (PSAD) <0.15 ng/mL/cm^3^ (blue) and ≥0.15 ng/mL/cm^3^ (red). Calculations were performed using competing risk functions. (**A**) Plot showing both competing events. (**B**) Plot focusing solely on prostate cancer mortality.

We used competing risk functions to estimate cumulative prostate cancer and other‐cause mortality. We plotted cumulative prostate cancer mortality and other‐cause mortality stratified by PSAD for participants (Fig. 1). We reported cumulative incidences of prostate cancer mortality at 5, 10, 15 and 20 years and assessed the significance of differences between the strata using Grey's test (Appendix S1). As a *post hoc* analysis, we repeated the cumulative incidence calculation using a PSA threshold of 10 ng/mL (Appendix S1).

To assess the additional capacity of PSAD to predict prostate cancer mortality over PSA and the base variables (age at biopsy, CCI, DRE result, socioeconomic status, 5‐ARI usage, and family history), we calculated the Akaike information criterion (AIC) and *c*‐statistics for each multivariable Cox regression model and used likelihood ratio tests to estimate the significance of improvements in model fit. The base variables were selected for their availability and clinical relevance. After assessing for the most adequate transformation based on model fit (Appendix S2), we used three‐ and four‐knot restricted cubic spline transformation for PSA and PSAD, respectively, while CCI was included as a numerical variable. DRE result, socioeconomic status, and family history were modelled as categorical variables. To preserve degrees of freedom, we merged the categories ‘Suspicious’ and ‘Malignant’ for DRE status and regarded socioeconomic status ‘Student’ as missing data (Table 1). We imputed any missing values (Table 1) of the variables included among the base variables using the *transcan* function from *Hmisc* library. We tested the proportional hazards assumption of the variables in the Cox regression model using statistical tests, graphical assessment of scaled Schoenfeld residuals, and log–log survival curves (after PSAD‐based stratification), and found no indication of violation (Appendix S3).

**Fig. 2:**
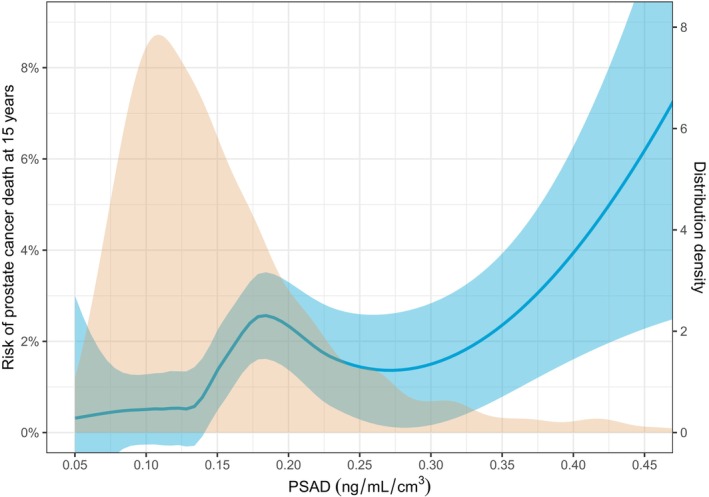
A non‐parametric regression model with locally estimated scatterplot smoothing illustrating the association of PSA density (PSAD) with prostate cancer mortality in men with a benign systematic TRUS‐guided prostate biopsy result. The distribution density plot of PSAD (yellow) is also shown on the second *y*‐axis on the right.

We used a non‐parametric regression model with locally estimated scatterplot smoothing (LOESS) to determine the association of PSAD with prostate cancer mortality (Fig. 2). Finally, we conducted a decision curve analysis to assess the clinical value of both continuous (transformed with restricted cubic spline) and dichotomous PSAD and PSA (PSAD threshold 0.15 ng/mL/cm^3^; PSA threshold 10 ng/mL) in predicting 15‐year prostate cancer mortality (Appendices S4 and S5).

As a post hoc analysis, we evaluated the impact of changes in biopsy protocols that occurred throughout the follow‐up period. Specifically, we compared the predictive performance of the multivariable Cox regression model consisting of the base model, PSA and PSAD among men stratified by the number of biopsy cores obtained at the time of the initial biopsy (≤6 cores vs > 6 cores). Additionally, we assessed whether including the number of obtained biopsy cores in the model (as a continuous variable or dichotomous variable using a threshold of six cores) would improve its performance in predicting prostate cancer mortality using previously described methodology.

A level of 5% was used for statistical significance. All reported *P* values are two‐sided and 95% CIs are presented for the main results. We did not conduct a power calculation for this study because no sampling was used. All analyses were performed using R Statistical Software 2023.06.2 Build 561 for MacOS (RStudio, PBC; 2022; Boston, MA, USA).

## Results

**Fig. 3:**
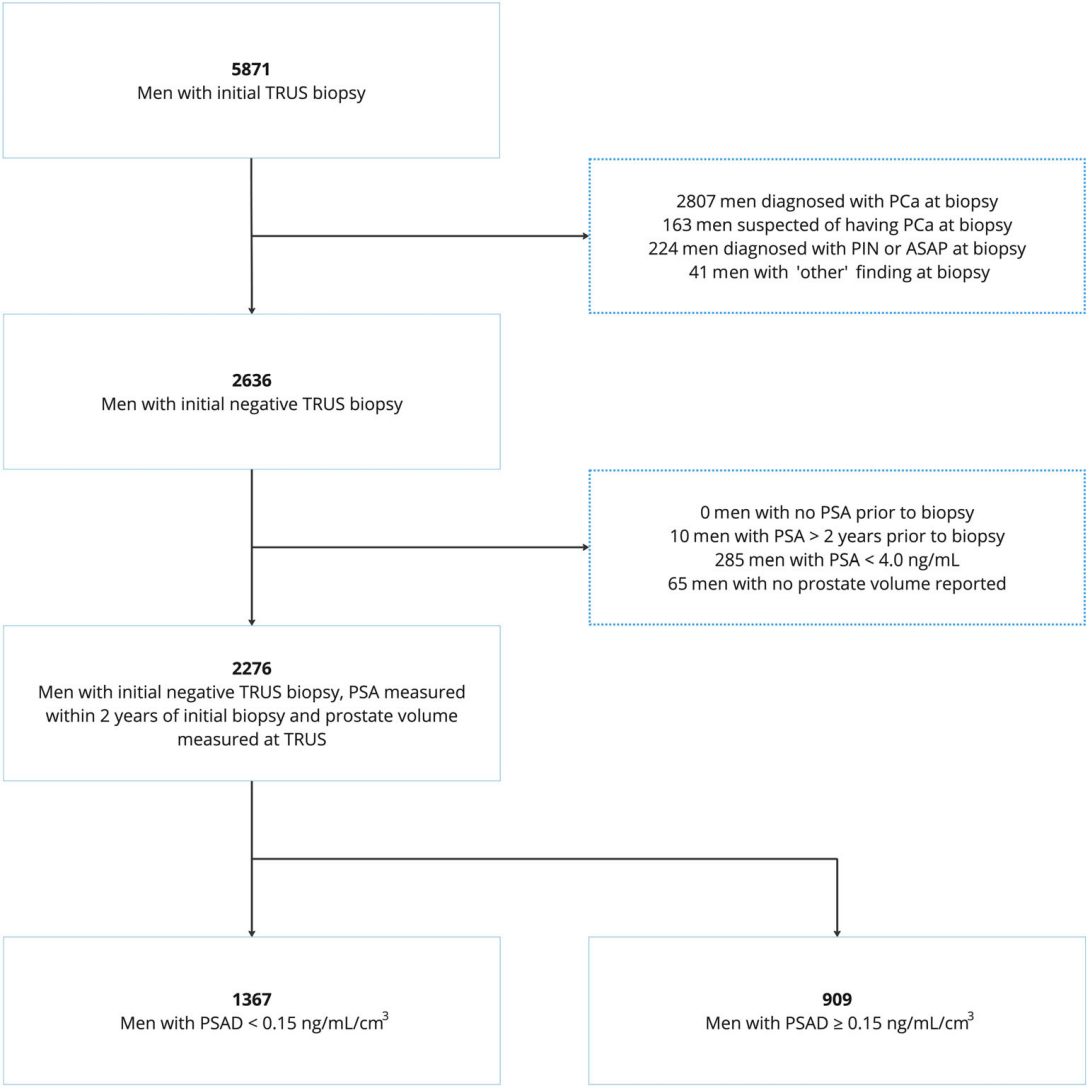
Flowchart of the study cohort selection. ASAP, atypical small acinar proliferation; PCa, prostate cancer; PIN, prostatic intraepithelial neoplasia; PSAD, PSA density.

During the three screening rounds, 5871 men underwent a systematic TRUS‐guided biopsy due to a PSA level ≥4 ng/mL. Of these, 2276 men with an initial benign biopsy result and calculable PSAD were eligible for analysis (Fig. 3).

The median (interquartile range [IQR]) age at the initial benign systematic TRUS‐guided biopsy was 63.6 (60.1–67.3) years. The median (IQR) follow‐up was 17.4 (13.2–20.9) years. The follow‐up for those without death from prostate cancer was 17.5 (13.2–21.0) years. There were 50 deaths from prostate cancer and 1028 from other causes. The median (IQR) age at prostate cancer death was 79 (75–84) years, and at death from other causes it was 78 (73–83) years. After the initial benign biopsy, 1260 men (55%) had a subsequent repeat systematic TRUS‐guided biopsy, resulting in 387 (17%) diagnoses of Gleason score ≤6 and 236 (10%) diagnoses of Gleason score ≥7 prostate cancer. The Gleason score information was unavailable for 83 men (3.6%) who were diagnosed with the disease. Of the men who died from prostate cancer, 34 (68%) had Gleason score ≥7 and 13 (26%) had Gleason score ≤6 disease, while two (4%) lacked Gleason score data, and for one man the diagnosis was made posthumously at autopsy. The median (IQR) interval from prostate cancer diagnosis to prostate cancer death was 7.7 (4.5–12) years and from initial biopsy to prostate cancer death it was 15 (11–18) years. Of the included men, 1367 (60%) had a PSAD <0.15 ng/mL/cm^3^. Detailed clinical characteristics and follow‐up results stratified by PSAD subgroup are presented in Table 1.

As demonstrated by the cumulative incidence plot (Fig. 1), among men with a benign systematic TRUS‐guided biopsy and PSA level ≥4 ng/mL, a PSAD threshold of 0.15 ng/mL/cm^3^ was associated with prostate cancer mortality (*P* < 0.001), but not with other‐cause mortality (*P* = 0.74; Fig. 1). The overall 15‐year prostate cancer mortality was 1.1% (95% CI 0.75%–1.6%). The 15‐year prostate cancer mortality was substantially lower in men with PSAD <0.15 ng/mL/cm^3^ (0.54% [95% CI 0.24%–1.1%]) than in men with PSAD ≥0.15 ng/mL/cm^3^ (2.0% [95% CI 1.2%–3.1%]). The 5‐, 10‐, 15‐ and 20‐year cumulative mortality results stratified by a PSA threshold of 10 ng/mL and a PSAD threshold of 0.15 ng/mL/cm^3^ are presented in Appendix S1. The association between PSAD and prostate cancer‐specific death was nonlinear and non‐monotonous (Fig. 2). However, the small number of prostate cancer deaths resulted in wide CIs with high PSAD.

The *c*‐statistic of Cox regression analysis utilising the base variables in predicting prostate cancer mortality was 0.737 (AIC 699.3). The discrimination was improved when PSA was added (*c*‐statistic 0.765, AIC 695.1; *P* = 0.016). Further adding PSAD to the model resulted in the best discriminative ability (*c*‐statistic 0.781, AIC 692.7) with a highly significant improvement over the base variables alone (*P* = 0.003) and modest improvement over the base variables and PSA (*P* = 0.039). However, the base variables combined with PSAD produced the best fit (AIC 691.2) and a similar *c*‐statistic (0.779) to the model that also included PSA. More detailed results of the Cox regression analysis are shown in Appendices S6 and S7.

Use of PSA and/or PSAD did not substantially contribute to prediction of other‐cause mortality (*c*‐statistic ranged 0.663–0.666 for all analysis).

Incorporating the number of biopsy cores, whether as a continuous (*c*‐statistic 0.779, AIC 693.5; *P* = 0.28) or dichotomous variable (*c*‐statistics 0.781, AIC 692.8; *P* = 0.17), into the Cox regression model that included the base variables, PSA and PSAD (*c*‐statistics 0.781; AIC 692.7) did not improve the ability to predict prostate cancer mortality. However, the model consisting of the base variables, PSA and PSAD performed substantially better for the subgroup of men who underwent biopsies with >6 cores compared to those with ≤6 cores (*c*‐statistics 0.866 vs 0.766).

Finally, the decision curve analysis (Appendix S4) demonstrated that PSAD‐based stratification using a threshold of 0.15 ng/mL/cm^3^ provides net benefit over alternative strategies at low‐risk threshold levels (0.9%–1.8% vs PSA ≥10 ng/mL, and ‘intervention for all’ and ‘intervention for none’ strategies). The decision curve analysis (Appendix S5) using continuous PSA and PSAD transformed using three‐ and four‐knot restricted cubic splines produced similar results.

## Discussion

The biomarker PSAD is a widely employed prostate cancer risk indicator, but its association with mortality has not been properly evaluated previously [12, 18, 19, 20, 21]. Our results, with a median follow‐up of 17.6 years, demonstrate that PSAD predicts prostate cancer death in men with a benign systematic TRUS‐guided prostate biopsy result. Moreover, our findings indicate that risk stratification based on PSAD provides information in addition to PSA and other clinical factors including age, DRE result, family history, and CCI score. Integrating PSAD‐based risk assessment into the decision‐making process could aid in determining the appropriate follow‐up strategy.

In accordance with recent screening and population‐based studies, however, long‐term prostate cancer mortality after a benign systematic TRUS‐guided biopsy result was very low. The observed 1.1% 15‐year prostate cancer mortality rate is similar to the 1.1%–1.3% prostate cancer mortality rates found in United States [6] (1.3%) and Canadian [4] (1.1%) populations, but is notably lower than the 1.9% prostate cancer mortality rate in a recent Danish cohort [2]. Conversely, the 2.1% 20‐year prostate cancer mortality rate in our study is higher than that in studies from the United States [6], Canada [4] and Sweden [5], where mortality ranged from 1.4% to 1.8%.

While the variations in prostate cancer mortality have multifactorial causes, several studies have associated higher screening intensity with greater reduction in prostate cancer mortality. Unorganised opportunistic PSA testing without rigorous follow‐up has been found to be less effective than organised screening in reducing prostate cancer mortality [22, 23]. In the Finnish arm of the ERSPC trial, a less intensive screening protocol was utilised compared with the Swedish and Dutch arms, which also reported greater mortality reductions [24]. Similarly, of the three screening‐based studies evaluating 20‐year prostate cancer mortality after a benign systematic TRUS‐guided biopsy result, our study had the lowest screening frequency (quadrennial vs biannual and annual) and the highest prostate cancer mortality (2.1% vs 1.4%–1.6%) [5, 6]. Furthermore, out of the studies that have previously evaluated prostate cancer mortality after a benign systematic TRUS‐guided biopsy and reported re‐biopsy rates, the population‐based Danish study had the lowest re‐biopsy rate (17% vs 32%) and the highest 15‐year prostate cancer mortality (1.9% vs 1.1–1.1%) [2, 4].

These findings imply that some individuals would benefit from more intensive monitoring after a benign systematic TRUS‐guided biopsy result. However, increased monitoring without proper patient selection would also lead to increased overdiagnosis and overtreatment. Hence, patient stratification with reflex tests is needed. Studies assessing long‐term prostate cancer mortality after a benign systematic TRUS‐guided biopsy result have shown that PSA is associated with prostate cancer mortality even in such a low‐risk population [2, 4, 6]. However, these studies were unable to assess the association of prostate cancer mortality with PSAD. PSAD can predict subsequent Gleason score ≥7 prostate cancer after a benign systematic TRUS biopsy better than PSA and our study is the first to demonstrate that this extends to mortality [12].

The advances in acquisition and reporting of prostate MRI since the launch of the Prostate Imaging‐Reporting and Data System (PI‐RADS) in 2012 [25], and its major revision in 2015 [26], have caused a paradigm shift in prostate cancer diagnostics. MRI has been shown to provide better risk stratification than conventional TRUS‐guided biopsies, with improved sensitivity and specificity for Gleason ≥7 prostate cancer [27]. Together with the lessons learned from the challenges related to PSA and TRUS‐guided biopsy‐based screening, these advances have caused a shift towards ideology that emphasises individual risk adaption [1]. This transition has been facilitated by the PSA‐derived reflex tests (such as 4KScore, Prostate Health Index, and Stockholm3 test) that offer another layer of stratification prior to biopsy. The major advantage of PSAD as a reflex test is the ability to calculate it as a by‐product of the current standard of care – from a single blood sample and volume estimation, measured on TRUS or MRI. Hence, it is globally available at marginal additional cost.

Although PSAD has been associated with the risk of subsequent Gleason score ≥7 prostate cancer after a benign MRI result [18, 20, 21, 28], because of the relative novelty of MRI and the typically slow progression of prostate cancer, the capacity of PSAD to predict mortality in MRI‐negative men remains unclear. Given the median intervals of 7.7 years from prostate cancer diagnosis to disease‐specific death and 15 years from the initial biopsy to disease‐specific death in our study, follow‐up spanning at least a decade is needed to accumulate such data. However, our findings demonstrate the ability of PSAD to predict prostate cancer mortality among men with low‐risk disease, and the results are likely also applicable in the context of MRI‐based diagnostics.

Our decision to use a PSAD threshold of 0.15 ng/mL/cm^3^ was based on its frequent application in the previous literature for estimating the risk of Gleason score ≥7 prostate cancer following a benign MRI [18]. The European Association of Urology guidelines recognise a PSAD below 0.15 ng/mL/cm^3^ as a hallmark of low‐risk prostate cancer in men undergoing active surveillance [1]. Furthermore, this threshold has been widely adopted in Finland for clinical practice, serving as a trigger for biopsies. However, no consensus on the optimal threshold for PSAD exists. Recent research has suggested that a higher threshold (0.20 ng/mL/cm^3^) should be used after a benign MRI result, unless the MRI images are of suboptimal quality [29]. Contrary to our results, Pellegrino et al. [29] found no discontinuity of Gleason ≥7 prostate cancer risk around PSAD 0.15 ng/mL/cm^3^. In our study, the LOESS analysis showed a local peak in prostate cancer mortality at a PSAD of approximately 0.18 ng/mL/cm^3^ (Fig. 2). Hence, using a threshold of 0.20 ng/mL/cm^3^ would have resulted in poorer discrimination. However, this peak may be affected by cognitive (i.e., usage of PSA‐based risk thresholds) and sampling bias (i.e., low rate of prostate cancer death) and one should be cautious about drawing conclusions on the optimal PSAD thresholds based on our results alone.

In the end, one threshold will not suit everyone. The choice of threshold depends on multiple factors such as age, comorbidity burden, and personal preference – i.e., whether it is more important to avoid missing a prostate cancer or to avoid unnecessary diagnosis. This rationale extends to the use at all of PSAD as a reflex test. Our decision curve analysis demonstrates that, while PSAD provides net benefit over alternative strategies at low‐risk threshold levels, PSA performs better at higher threshold levels. Hence, neither biomarker is unequivocally superior – it is a matter of context‐specific preference.

Our study has some limitations. Our follow‐up data for prostate cancer diagnosis extended only up to 2016, while the mortality data were available until 2020. Despite our large study cohort, the relatively small numbers of prostate cancer deaths resulted, in some cases, in wide CIs and high variance (e.g., the LOESS plot for association of prostate cancer‐specific death and PSAD).

It is also important to acknowledge that clinical practices have evolved over the nearly two‐decade follow‐up period. In the FinRSPC study, a transition from a 6‐ to a 10‐ to 12‐core prostate biopsy protocol occurred in 2002. As a result, 70% of the men in our study were classified as negative for prostate cancer based on sextant biopsy results (Table 1), which may have caused sampling bias, as it is considered less accurate than the 10‐ to 12‐core biopsy protocol. Our post hoc analysis demonstrates that the number of biopsy cores at the time of the initial biopsy does not add predictive value to the model consisting of the base variables, PSA, and PSAD. However, the model consisting of the base variables, PSA, and PSAD predicts prostate cancer mortality substantially better for men with >6 biopsy cores at the initial biopsy than for men with ≤6 biopsy cores (*c*‐statistics 0.866 vs 0.766). This difference may reflect the superiority of updated biopsy and grading protocols, but it could also be related to differences in follow‐up duration, which was shorter for the men who underwent 12‐core biopsies.

Given that the median times to disease‐specific death from initial biopsy and from cancer diagnosis were 15 and 7.7 years, respectively, it is also possible that a portion of lethal prostate cancer cases in our study represents newly developed disease that would have been undetectable at the time of the initial biopsy, even if no sampling error had occurred. Lastly, a grade shift in Gleason score over time is a well‐known phenomenon. As Gleason scoring has been refined over time, some cases previously classified as Gleason score ≤6 prostate cancer could be considered Gleason score ≥7 by current standards [30]. While interpreting our results, it is also important to note that our data did not provide the highest prostate cancer grade found during the follow‐up period but only the grade at the time of the initial prostate cancer diagnosis.

Our study has several strengths. Although retrospective, the data were gathered as a part of a randomised screening trial (FinRSPC) with meticulous data collection. The data source of the main outcome (prostate cancer death) was the Finnish Causes of Death registry, which has previously been shown to possess excellent accuracy. A study comparing the official cause‐of‐death certificate data with an independent expert review in the FinRSPC population found excellent overall agreement (kappa 0.95), with 96.1% sensitivity and 98.9% specificity in identifying prostate cancer deaths [31]. Likewise, the Finnish Cancer Registry has 96% coverage of solid tumours [14]. Most importantly, the follow‐up time in our study was long enough to demonstrate the association of PSAD with prostate cancer mortality.

In conclusion, prostate cancer mortality after a benign systematic TRUS‐guided biopsy is very low. In this population, PSAD predicts prostate cancer mortality, and improves prognostic prediction when combined with PSA. PSAD‐based stratification can be used to guide follow‐up strategy but additional research is needed to assess whether these findings apply to men with a benign MRI result.

## Funding

This work was supported in part by a grant from the Cancer Foundation Finland (to A. Rannikko; Grant number 180141), Competitive State Research Funding (VTR) administered by HUS Helsinki University Hospital (to Prof. Rannikko; Grant number TYH2021332), the Jane and Aatos Erkko Foundation (to A. Rannikko on 29.5.2020) and the Research Council of Finland Grant no. 260931 (A. Auvinen). Open access funded by Helsinki University Library. The funding organisations had no role in the design and conduct of the study, the collection, management, analysis, or interpretation of the data, the preparation, review, or approval of the manuscript, nor the decision to submit the manuscript for publication.

## Disclosure of Interests

A. Rannikko is a member of the board of the Ida Montin Foundation and Orion Research Foundation, an advisory board member for medical companies Bayer, Orion Pharma and Janssen, a clinical advisor for Aqsens company in which he has stock, and an investigator in clinical trials by Rho‐Vac, Orion Pharma, Bayer, Astellas, Pfizer and Janssen. J. Pylväläinen, A. Auvinen, J. Raitanen and K. Talala have no conflicts of interest.

## Author Contributions

Ms Talala had full access to all the data in the study and takes responsibility for the integrity of the data. Mr Pylväläinen had limited access to the data and takes responsibility for the accuracy of the data analysis. Concept and design: Pylväläinen, Talala, Auvinen, Rannikko. Acquisition, analysis, or interpretation of data: All authors. Drafting of the manuscript: Pylväläinen. Critical revision of the manuscript for important intellectual content: All authors. Statistical analysis: Pylväläinen, Raitanen, Auvinen. Obtained funding: Rannikko. Administrative, technical, or material support: Talala, Raitanen, Auvinen. Supervision: Talala, Raitanen, Auvinen, Rannikko.

## Supporting information


**Appendix S1.** 5‐, 10‐, 15‐ and 20‐year cumulative prostate cancer mortality rates stratified by PSA cutoff 10 ng/mL and PSAD cutoff 0.15 ng/mL/cm^3^.
**Appendix S2.** Table showing model fit (AIC) for multivariable Cox regression models consisting of the base variables and various transformations of PSA and PSAD.
**Appendix S3.** Results of testing the proportional hazards assumption of the multivariable Cox regression model consisting of the base variables, PSA, and PSAD, using statistical (A) and graphical (B, C) assessments of Schoenfeld residuals. Only the plots of scaled Schoenfeld residuals for total PSA and PSAD are shown (B, C). (D) Shows the result of the proportional hazards assumption test using log‐log survival curves (PSAD ≥0.15 ng/mL/cm^3^ vs PSAD <0.15 ng/mL/cm^3^).
**Appendix S4.** Decision curve analysis evaluating the net benefit of Cox regression model fit with dichotomous PSA (red) using threshold 10 ng/mL and PSAD (blue) using threshold 0.15 ng/mL/cm^3^ and compared to ‘intervention to all’ (dark blue) and ‘intervention to none’ (yellow) strategies in assessing the risk of prostate cancer mortality.
**Appendix S5.** Decision curve analysis evaluating the net benefit of Cox regression model fit with continuous PSA (red) and PSAD (blue) transformed with RCS compared to ‘intervention to all’ (dark blue) and ‘intervention to none’ (yellow) strategies in assessing the risk of prostate cancer mortality.
**Appendix S6.** Results of Cox proportional hazards regression showing hazard ratios (excluding nonlinear variables) and *P* values for all variables.
**Appendix S7.** Graphical presentation of the Cox proportional hazards regression model for all variables.

## Data Availability

The datasets generated during and/ or analysed during the current study are not publicly available due to national law and data permit requirements.
