# Supplementary material for: Association of prostate‐specific antigen density with prostate cancer mortality after a benign systematic prostate biopsy result
Source: BJU Int. 2025 Jan 22;135(5):841–50. doi: 10.1111/bju.16641 (PMC11975165; doi:10.1111/bju.16641)

# Appendix

Appendix 1. 5-, 10-, 15- and 20-year cumulative prostate cancer mortality rates stratified by PSA cutoff 10 ng/mL and PSAD cutoff 0.15 ng/mL/cm^3^.

| **Characteristic** | **5-year mortality, % (95% CI)** | **10-year mortality, % (95% CI)** | **15-year mortality, % (95% CI)** | **20-year mortality, % (95% CI)** | ***P* value***^a^* |
| --- | --- | --- | --- | --- | --- |
| Overall | 0.13 (0.04–0.37) | 0.40 (0.20–0.73) | 1.1 (0.75–1.6) | 2.1 (1.5–2.8) |  |
| PSA |  |  |  |  | < 0.001 |
| *< 10 ng/mL* | 0.05 (0.01–0.27) | 0.29 (0.12–0.61) | 0.89 (0.55–1.4) | 1.8 (1.3–2.5) |  |
| *≥ 10 ng/mL* | 1.1 (0.22–3.6) | 1.7 (0.45–4.4) | 3.9 (1.7–7.5) | 5.9 (3.0–10) |  |
| PSAD |  |  |  |  | 0.001 |
| *< 0.15 ng/mL/cm^3^* | 0.00 (NA) | 0.15 (0.03–0.51) | 0.54 (0.24–1.1) | 1.3 (0.75–2.1) |  |
| *≥ 0.15 ng/mL/cm^3^* | 0.33 (0.09–0.92) | 0.77 (0.35–1.5%) | 2.0 (1.2–3.1) | 3.3 (2.2–4.7) |  |
| Abbreviations: CI, confidence interval; NA, not applicable; PSA, prostate-specific antigen; PSAD, PSA density  *^a^* Gray’s Test | | | | | |

Appendix 2. Table showing model fit (AIC) for multivariable Cox regression models consisting of the base variables and various transformations of PSA, PSAD.

|  | **PSAD transformation** | | |
| --- | --- | --- | --- |
| **PSA transformation** | **3-knot RCS** | **4-knot RCS** | **5-knot RCS** |
| 3-knot RCS, AIC | 694.8 | **692.7** | 694.4 |
| 4-knot RCS, AIC | 694.8 | 693.6 | 695.2 |
| 5-knots RCS, AIC | 696.5 | 695.2 | 696.8 |
| Abbreviations: AIC, Akaike information criterion; PSA, prostate-specific antigen; PSAD, PSA density; RCS, restricted cubic spline | | | |

Appendix 3. Results of testing the proportional hazards assumption of the multivariable Cox regression model consisting of the base variables, PSA, and PSAD, using statistical (A) and graphical (B and C) assessments of Schoenfeld residuals. Only the plots of scaled Schoenfeld residuals for total PSA and PSAD are shown (B and C). Figure D shows the result of the proportional hazards assumption test using log-log survival curves (PSAD ≥ 0.15 ng/mL/cm^3^ vs. PSAD < 0.15 ng/mL/cm^3^).

A

|  | Chi-squared | Degrees of freedom | *P* value |
| --- | --- | --- | --- |
| Age at diagnosis | 0.0829 | 1 | 0.77 |
| CCI | 0.3563 | 1 | 0.55 |
| Family history | 0.5030 | 1 | 0.48 |
| Socioeconomic group | 3.3830 | 5 | 0.64 |
| DRE result | 0.0089 | 1 | 0.92 |
| 5-ARI usage | 0.9259 | 1 | 0.34 |
| Total PSA | 0.8544 | 2 | 0.64 |
| PSAD | 2.2747 | 3 | 0.51 |
| **Global** | 10.1110 | 15 | 0.81 |
| Abbreviations: 5-ARI = 5α-reductase inhibitor; CCI, Charlson Comorbidity Index; DRE, digital rectal examination; PSA, prostate-specific antigen; PSAD, PSA density; TRUS, transrectal ultrasound. | | | |

B


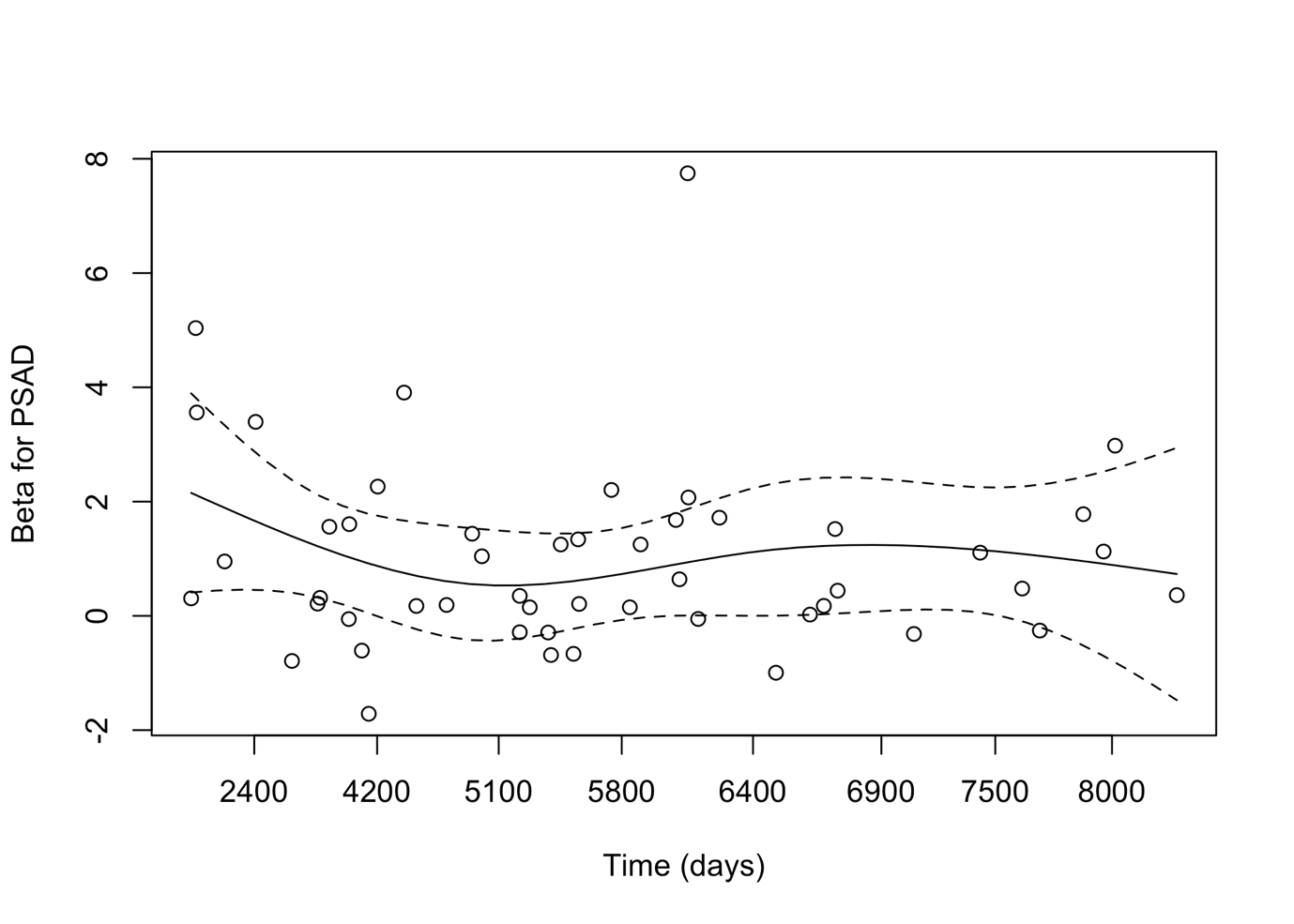


C


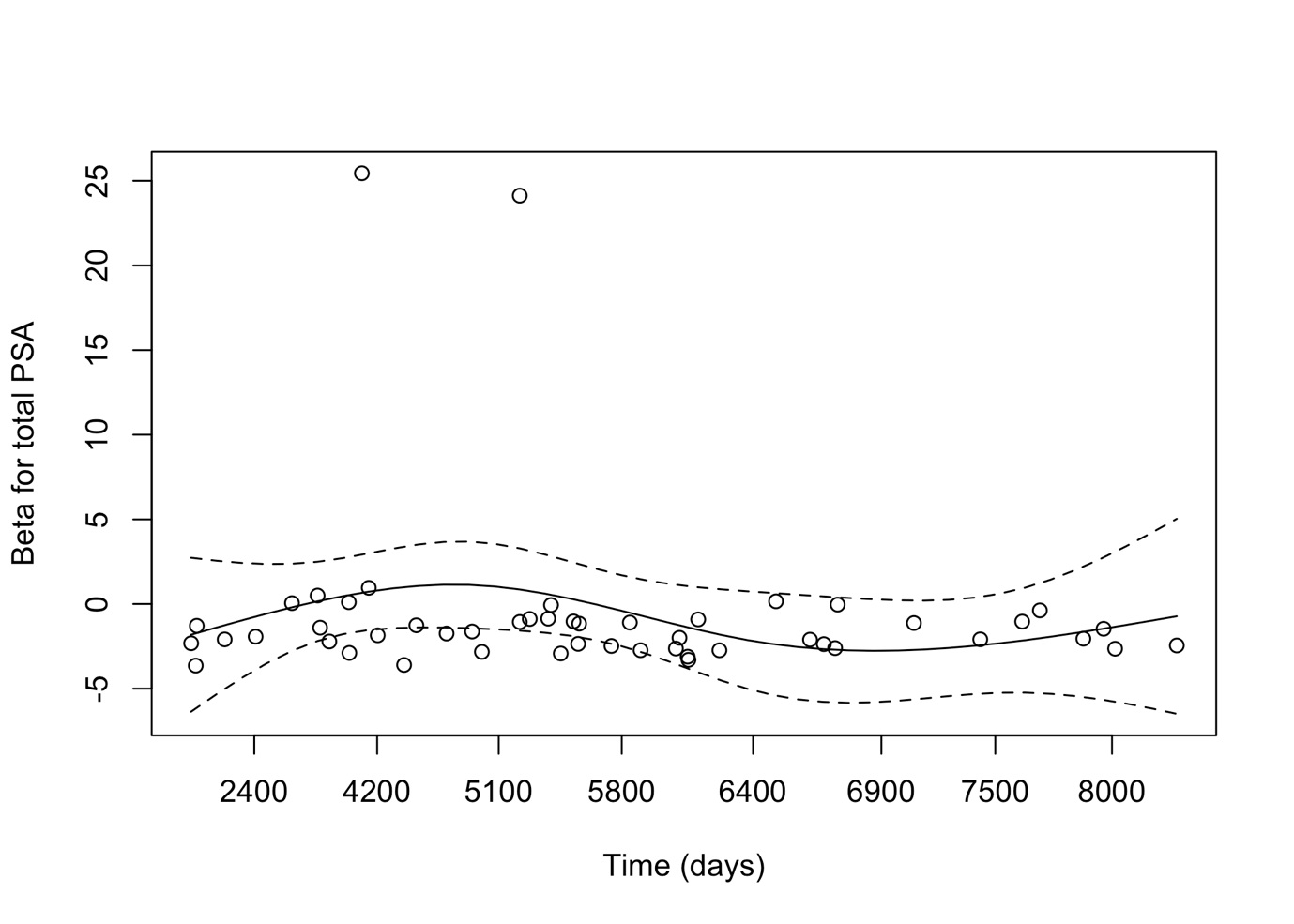


D


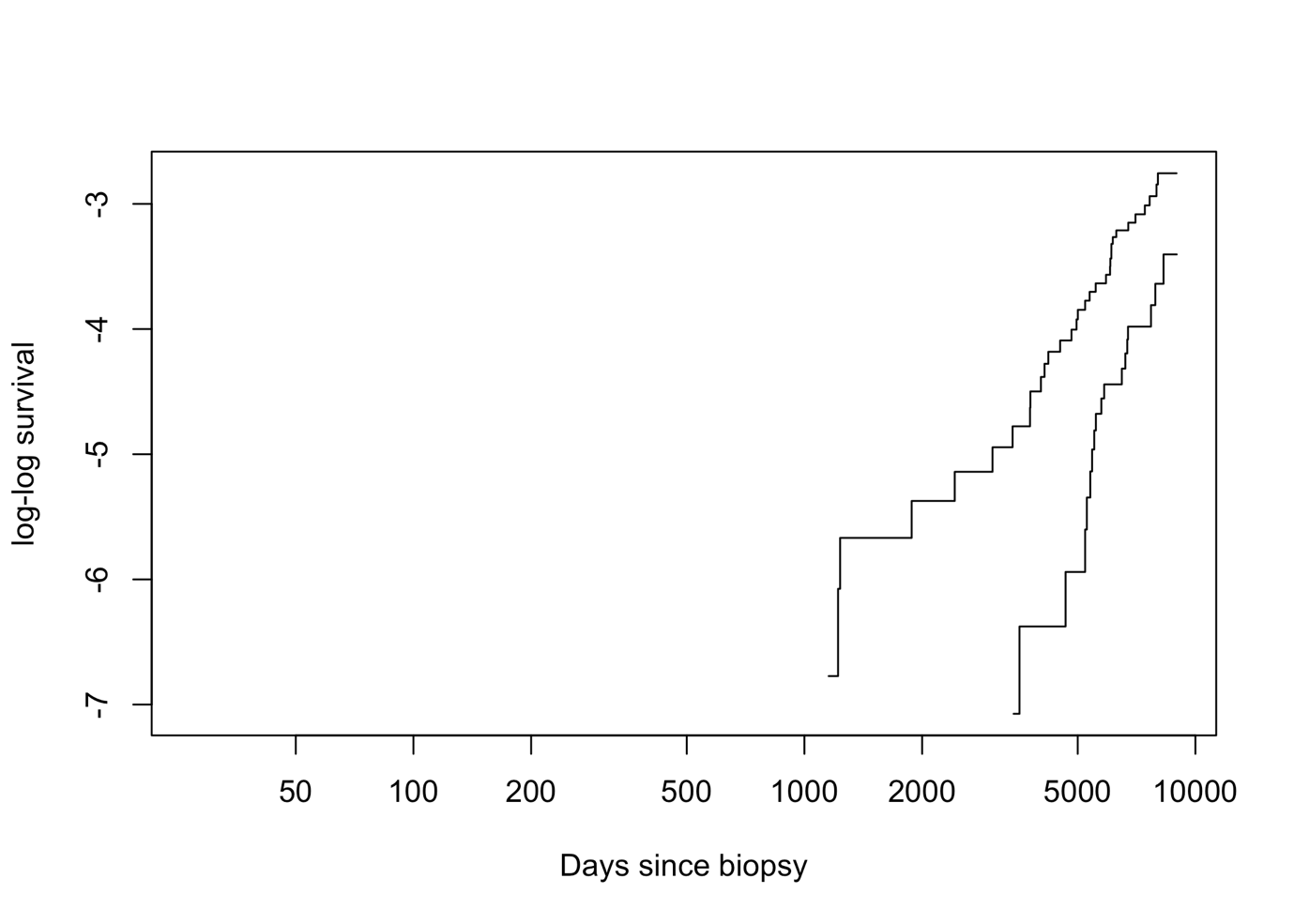


Appendix 4. Decision curve analysis evaluating the net benefit of Cox regression model fit with dichotomous PSA (red) using threshold 10 ng/mL and PSAD (blue) using threshold 0.15 ng/mL/cm^3^ and compared to “intervention to all” (dark blue) and “intervention to none” (yellow) strategies in assessing the risk of prostate cancer mortality.


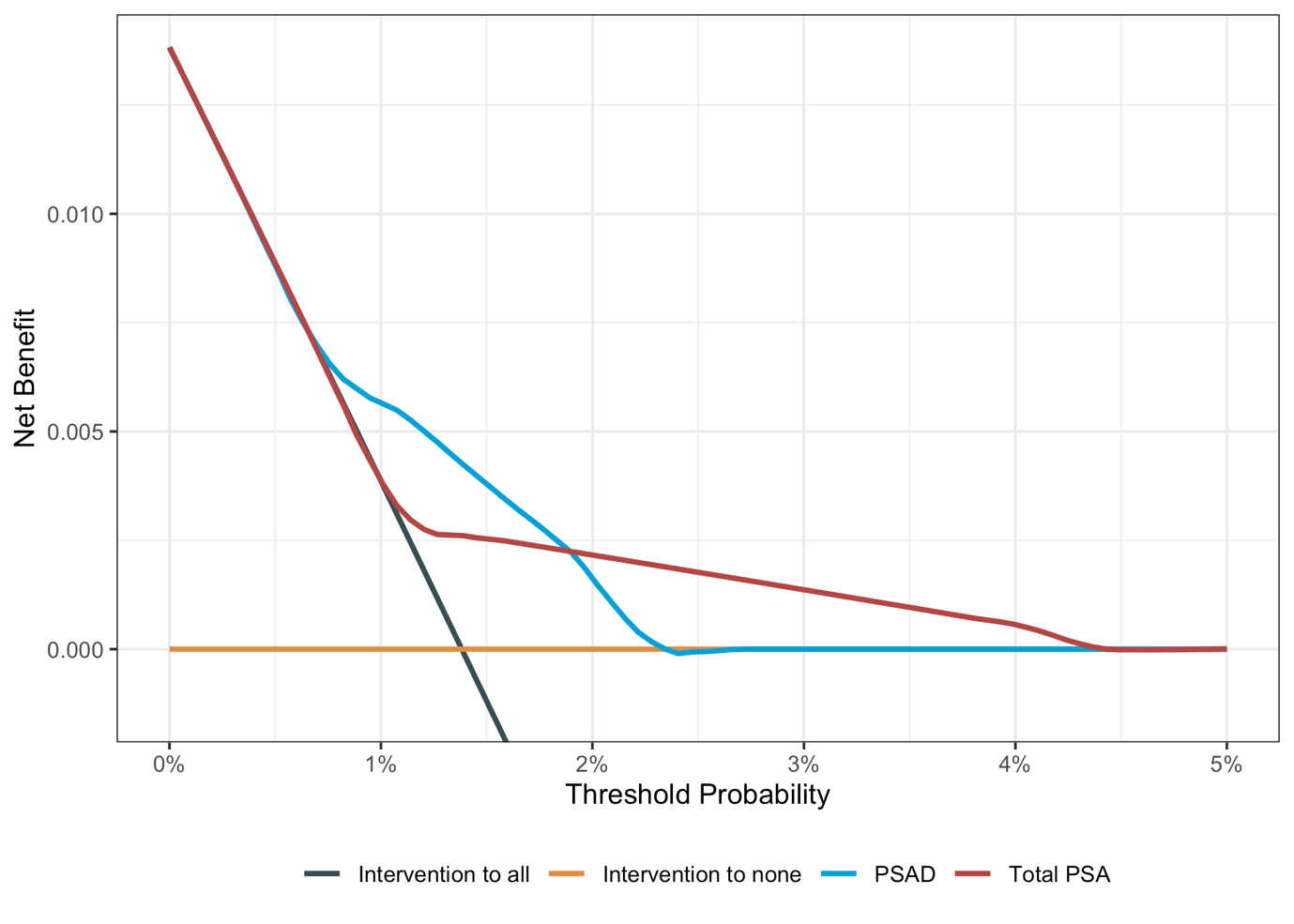


Appendix 5. Decision curve analysis evaluating the net benefit of Cox regression model fit with continuous PSA (red) and PSAD (blue) transformed with RCS compared to “intervention to all” (dark blue) and “intervention to none” (yellow) strategies in assessing the risk of prostate cancer mortality.


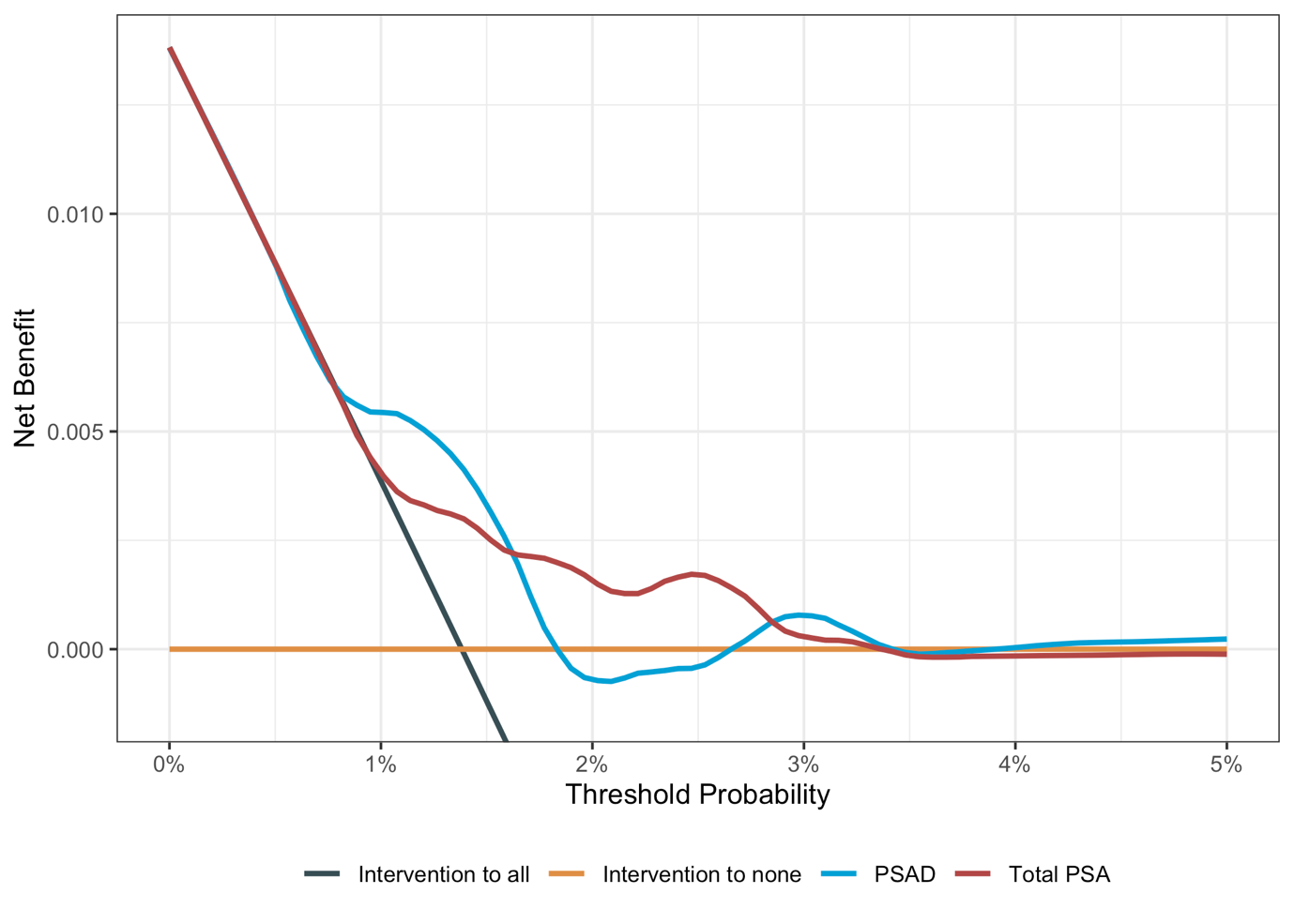


Appendix 6. Results of Cox proportional hazards regression showing hazard ratios (excluding nonlinear variables) and *P* values for all variables.

| Characteristic | HR | 95% CI | *P* value |
| --- | --- | --- | --- |
| Age at initial biopsy | 1.07 | 0.99–1.15 | 0.10^a^ |
| CCI | 1.07 | 0.51–2.24 | 0.9 |
| Family history |  |  |  |
| *No family history* | Ref. | NA |  |
| *1st relative prostate cancer* | 1.61 | 0.68–3.82 | 0.3 |
| Socioeconomic status |  |  |  |
| *Upper level employees* | Ref. | NA |  |
| *Manual workers* | 0.53 | 0.11–2.62 | 0.4 |
| *Lower level employees* | 0.80 | 0.20–3.20 | 0.8 |
| *Self-employed person* | 0.35 | 0.04–3.29 | 0.4 |
| *Pensioner* | 1.60 | 0.62–4.10 | 0.3 |
| *Unemployed* | 3.27 | 1.18–9.08 | 0.023 |
| DRE result |  |  |  |
| *Normal* | Ref. | NA |  |
| *Suspicious or Malignant* | 3.72 | 2.02–6.91 | < 0.001 |
| 5-ARI usage |  |  |  |
| *No* | Ref. | NA |  |
| *Yes* | 0.54 | 0.12–2.37 | 0.3^a^ |
| Total PSA (3-knot RCS) | NA^b^ | NA^b^ | 0.3^a^ |
| PSAD (4-knot RCS) | NA^b^ | NA^b^ | 0.038^a^ |
| Abbreviations: 5-ARI = 5α-reductase inhibitor; CCI, Charlson Comorbidity Index; CI, Confidence Interval; DRE, digital rectal examination; HR, Hazard Ratio; PSA, prostate-specific antigen; PSAD, PSA density; RCS, Restricted Cubic Spline  ^a^ Likelihood-ratio test; ^b^ No HR calculated for nonlinear variables | | | |

Appendix 7. Graphical presentation of the Cox proportional hazards regression model for all variables. The outcome event is prostate cancer survival.


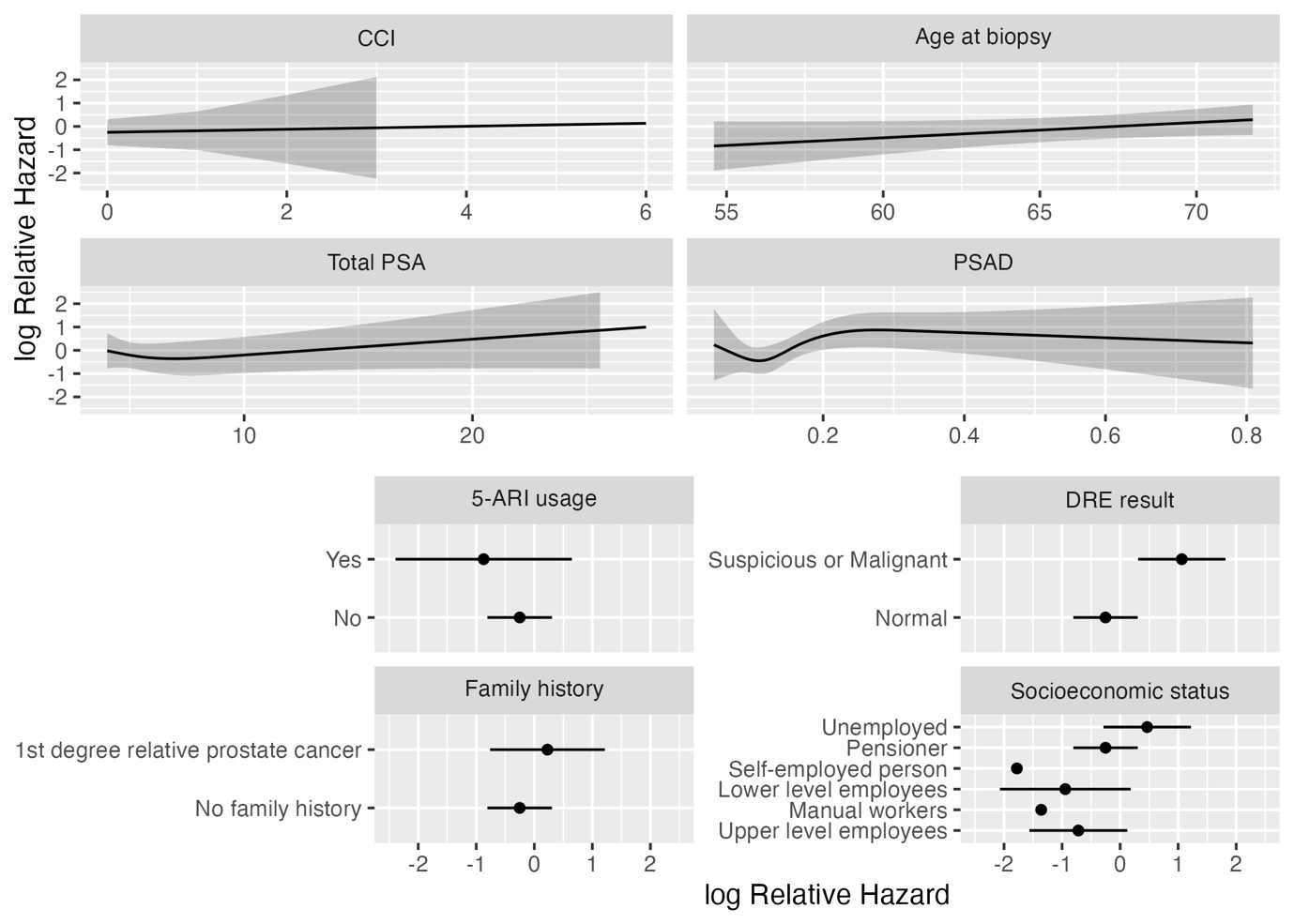

Supplement: Supplementary file 1 — Appendix S1. 5‐, 10‐, 15‐ and 20‐year cumulative prostate cancer mortality rates stratified by PSA cutoff 10 ng/mL and PSAD cutoff 0.15 ng/mL/cm3. Appendix S2. Table showing model fit (AIC) for multivariable Cox regression models consisting of the base variables and various transformations of PSA and PSAD. Appendix S3. Results of testing the proportional hazards assumption of the multivariable Cox regression model consisting of the base variables, PSA, and PSAD, using statistical (A) and graphical (B, C) assessments of Schoenfeld residuals. Only the plots of scaled Schoenfeld residuals for total PSA and PSAD are shown (B, C). (D) Shows the result of the proportional hazards assumption test using log‐log survival curves (PSAD ≥0.15 ng/mL/cm3 vs PSAD <0.15 ng/mL/cm3). Appendix S4. Decision curve analysis evaluating the net benefit of Cox regression model fit with dichotomous PSA (red) using threshold 10 ng/mL and PSAD (blue) using threshold 0.15 ng/mL/cm3 and compared to ‘intervention to all’ (dark blue) and ‘intervention to none’ (yellow) strategies in assessing the risk of prostate cancer mortality. Appendix S5. Decision curve analysis evaluating the net benefit of Cox regression model fit with continuous PSA (red) and PSAD (blue) transformed with RCS compared to ‘intervention to all’ (dark blue) and ‘intervention to none’ (yellow) strategies in assessing the risk of prostate cancer mortality. Appendix S6. Results of Cox proportional hazards regression showing hazard ratios (excluding nonlinear variables) and P values for all variables. Appendix S7. Graphical presentation of the Cox proportional hazards regression model for all variables. [file BJU-135-841-s001.docx]
